# Food Addiction and Its Relationship to Weight- and Addiction-Related Psychological Parameters in Individuals With Overweight and Obesity

**DOI:** 10.3389/fpsyg.2021.736454

**Published:** 2021-09-21

**Authors:** Magdalena Pape, Stephan Herpertz, Stefanie Schroeder, Caroline Seiferth, Tanja Färber, Jörg Wolstein, Sabine Steins-Loeber

**Affiliations:** ^1^Department of Psychosomatic Medicine and Psychotherapy, LWL-University Hospital, Ruhr University Bochum, Bochum, Germany; ^2^Department of Clinical Psychology and Psychotherapy, University of Bamberg, Bamberg, Germany; ^3^Department of Pathopsychology, University of Bamberg, Bamberg, Germany

**Keywords:** food addiction, obesity, overweight, eating disorders, eating behaviour, weight loss treatment

## Abstract

**Background and Aims:** It is assumed that a relevant subgroup of individuals experiences an addiction-like eating behaviour (Food Addiction), characterized by an impaired control over eating behaviour, emotional eating and food craving. Individuals experiencing Food Addiction partially share common symptomatology with Binge-Eating-Disorder and Bulimia Nervosa. The aim of this study was to investigate the prevalence of Food Addiction, general psychopathology, and associations with weight- and addiction-related constructs in individuals with overweight and obesity, who did not suffer from Binge-Eating-Disorder or Bulimia Nervosa.

**Methods:***N*=213 (67.1% female; M_BMI_=33.35kg/m^2^, SD_BMI_=3.79kg/m^2^) participants who were included in a weight loss program (I-GENDO project) reported BMI and completed questionnaires before the start of the treatment. Food Addiction severity, depressive symptoms, alcohol use disorder, internet use disorder, psychological distress, impulsivity personality trait, impulsive and emotional eating behaviour, food related inhibitory control, weight bias internalization, and self-efficacy were assessed.

**Results:** The prevalence of Food Addiction was 15% with higher, although not statistically significant, prevalence in female (18.2%) compared to male (8.6%) participants. Food Addiction was associated with higher BMI at baseline assessment, low self-esteem, impulsive and emotional eating behaviour, weight bias internalization, and deficits in food-related inhibitory control. In addition, correlations were found between Food Addiction and severity of depressive symptoms, internet use disorder, and psychological distress.

**Conclusion:** A relevant subgroup of participants experiences Food Addiction even when controlling for Binge-Eating-Disorder and Bulimia Nervosa. Future studies are warranted that investigate whether Food Addiction affects treatment success.

## Introduction

In the last decades, the prevalence of overweight and obesity has increased dramatically worldwide, causing not only physical, but also mental health problems, including depression and anxiety disorders (Chu et al., 2019). Various weight loss programs (WLPs) have been developed mainly based on physical activity, diets, and change in eating habits (Jensen and Ryan, 2014). These *lifestyle interventions* often lack long-term effectiveness (Jeffery et al., 2000; Ross Middleton et al., 2012) due to an “obesogenic environment” (Swinburn et al., 1999) that promotes gaining weight, and is not conducive to weight loss within the home or workplace (hypercaloric diet, physical inactivity). Furthermore, relevant individual psychological aspects associated with eating behaviour are not sufficiently considered (Chalk, 2004).

A subgroup of individuals with overweight and obesity describes themselves as being “addicted” to food, characterized by an impaired control over their eating behaviour, emotional eating and food craving (Davis et al., 2013; Meule, 2015). Firstly, the addiction model of overeating was introduced by self-help groups and is even today controversially discussed in the scientific community (Naish et al., 2018; Hebebrand and Gearhardt, 2021). In 2009, the Yale Food Addiction Scale (YFAS), the first formal measure of *Food Addiction* (FA), was introduced. Originally based on the DSM-IV criteria for diagnosing substance use disorders (SUD) the YFAS was adapted to addictive overeating (Gearhardt et al., 2009). With the updated criteria for addictive disorders in the DSM-5, the YFAS 2.0 was developed in 2016 (Gearhardt et al., 2016). Until now, FA is not formally included as a distinct category in DSM-5 or ICD-11, but is based on criteria for addictive disorders as defined in these diagnostic manuals.

The concept of FA gains support from neurobiological findings, reporting food related brain activities, i.e., in the dopaminergic reward system, similar to those of drug intake (Gearhardt et al., 2011; Grosshans et al., 2012; Beitscher-Campbell et al., 2016; Novelle and Diéguez, 2018). Consistent with neurobiological similarities, individuals experiencing FA share specific behavioural deficits and personality traits with individuals suffering from SUDs, such as increased impulsivity (VanderBroek-Stice et al., 2017). Impulsivity is a multidimensional construct characterized by deficient inhibitory processes and low self-control. There are mainly three components of impulsivity to be distinguished: *impulsive personality trait*, *impulsive actions*, i.e., deficits in behavioural inhibition (action withdrawal, action cancellation), and *impulsive choices*, i.e., the tendency towards immediate rewards instead of long-term goals (delay discounting; Hofmann et al., 2009; Bari and Robbins, 2013; Mole et al., 2015). Individuals experiencing FA report a tendency to consume immediate disposable high-caloric food, as well as problems in resisting eating impulses (Meule, 2017; VanderBroek-Stice et al., 2017; Preuss et al., 2019). In general, individuals experiencing FA are characterized by high impulsivity traits (Mobbs et al., 2010; Churchill and Jessop, 2011; Murphy et al., 2014). Individuals with overweight and obesity experiencing weight stigmatization are likely to internalize negative stereotypes and to attribute them to their own weight (weight bias internalization, WBI; Puhl and Heuer, 2009). WBI is assumed to be linked to emotional eating and FA, resulting in maladaptive eating patterns (Baldofski et al., 2016). FA is associated with low self-esteem, high psychological distress, lower quality of life and depressive symptoms (Gearhardt et al., 2012; Burmeister et al., 2013; Minhas et al., 2021; Vidmar et al., 2021). From a psychobiological perspective, there is the assumption that similar processes in FA and other addiction disorders may be operating, which might explain the similarities and the high comorbidity of FA with substance-related disorders and behavioural addictions (Davis and Carter, 2009; García-García et al., 2014; Marmet et al., 2019).

In Germany, about 7.9% of the general population suffer from FA, with increasing prevalence rates in individuals with overweight (17.2%; Hauck et al., 2017). According to a meta-analysis of *N*=25 studies, the prevalence of FA is higher in individuals with overweight and obesity (weighted mean prevalence, WMP: 25%), females (WMP: 12%) and individuals aged over 35years (WMP: 22%; Pursey et al., 2014). To date, numerous heterogeneous results about the effect of FA on body weight and the influence on the success of WLPs have been published. In general, FA symptom severity is associated with higher BMI (Pursey et al., 2014). Some studies report higher study attrition and lower effectiveness of WLPs in individuals experiencing FA, while other studies did not find differences to individuals who are not experiencing FA (Burmeister et al., 2013; Clark and Saules, 2013; Meule et al., 2015; Fielding-Singh et al., 2019). Yet, the prevalence of FA in WLP samples is high (up to 34,5–38%; Meule et al., 2015; Vidmar et al., 2021). At least partially, some authors explain the relationship between FA and weight history as a result of comorbid mental disorders, particularly eating disorders (Eichen et al., 2013; Lent et al., 2014; Chao et al., 2019). Prevalence rates of FA in adults with Binge-Eating-Disorder (BED) range from 40 to 50%, while in individuals with Bulimia Nervosa (BN) the prevalence rises over 80% (Gearhardt et al., 2014; De Vries and Meule, 2016). Additionally, the prevalence of eating disorders, i.e., BED (up to 30%), are also higher in participants undergoing weight loss interventions (Herpertz et al., 2006; Blaine and Rodman, 2007; Davis, 2015).

The high comorbidity may be due to the fact that BED, BN and FA share common mechanisms such as binge-eating and reward dysfunction (Burrows et al., 2018). Especially the emotional component (e.g., fear of not being able to stop and guilt surrounding binge eating episodes) of BED seems to be associated with increased FA severity (Burrows et al., 2017). Thus, FA overlaps with BN and BED (Gearhardt et al., 2013; Meule et al., 2014; Meule, 2019b). Yet, there are also dissimilarities, which distinguish FA from BED and BN (Schulte et al., 2020). When conceptualizing FA as a specific form of SUDs, highly palatable and processed foods act as drugs. Yet, individuals experiencing FA need not necessarily binge these drugs, but could also graze them throughout the day, similar to other SUDs. Additionally, some studies report specific food-related withdrawal and tolerance symptoms (Avena et al., 2008; Burger and Stice, 2012).

Taken together, despite its similarities to other SUDs FA is currently not an officially recognized diagnosis. One contentious issue in the recent debate about the need of an FA diagnosis is the mentioned overlap of FA symptoms with those of other eating disorders (Gearhardt and Hebebrand, 2021; Hebebrand and Gearhardt, 2021). In order to contribute to this question, we analysed FA in a sample of individuals with overweight and obesity participating at a 12-week long WLP, who did not suffer from BED or BN.

The aims of our study were:

1. To analyse the prevalence of FA in a sample of individuals with overweight and obesity seeking treatment, who did not suffer from BED or BN.
2. To analyse relationships between FA severity, eating behaviours, general psychopathology and weight- and addiction-related factors, such as impulsivity, food related inhibitory control, self-efficacy and weight bias internalization.
3. To further characterize the subgroup of FA in contrast to non-food-addicted (NFA) participants.

## Materials and Methods

### Procedure

The dataset of this manuscript is retrieved from the I-GENDO project (*Gender-sensitive Enhancement of Common Weight Loss Strategies for Overweight and Obesity*, ClinicalTrials.gov Identifier: NCT04080193) a multicentre randomized controlled trial to assess the effectivity of a gender sensitive individualized smartphone-based intervention to reduce weight. Interested individuals were recruited from August 2019 until August 2020 *via* newspaper articles, radio features and oral presentations at rehab centres. At the baseline assessment the participants completed questionnaires and reported their current weight (kg) and height (m), of which the individual BMI (kg/m^2^) was calculated.

### Participants

Table 1 displays the eligibility criteria of the I-GENDO project. Following the guidelines of the German Society for General and Visceral Surgery (DGAV) and the German Association for the Study of Obesity (DAG) individuals with obesity class III (BMI>39.9) suffer from a complex multifactorial framework of psychological, social and physical problems and are therefore recommended to undergo a bariatric surgery. In order to avoid potentially confounding effects, we excluded individuals with obesity class III from participation, but provided further support. During the recruitment process we screened for major depression with the PHQ-9 (Löwe et al., 2002). Interested subjects who scored above the cut-off for major depression (≥ 20) were excluded from participation (Kroenke et al., 2001). Furthermore, if suicidal ideation was reported, people were contacted and subsequently diagnosed *via* telephone with a structured interview to clarify suicidal intentions by experienced psychologists. When suicidal ideation could not be convincingly negated by clinical judgement, subjects were referred further and excluded from this study. In prior studies heavy drinking was consistently associated with gaining weight, which we expected could influence the effectiveness of the WLP (Traversy and Chaput, 2015). Using the Alcohol Use Disorders Identification Test (AUDIT, Saunders et al., 1993) subjects were excluded when the sum score of the AUDIT was 15 or more, suggesting hazardous alcohol consumption (Conigrave et al., 1995). In case of suspected eating disorders, as assessed by the Munich ED-Quest (Fichter et al., 2015), subjects were contacted and subsequently diagnosed by experienced psychologists using the German version of the Eating Disorder Examination (EDE), a clinical interview for the assessment of eating disorder specific psychopathology (Hilbert and Tuschen-Caffier, 2016). Individuals who were diagnosed with BN or BED were excluded from participation.

**Table 1:** Eligibility criteria of the I-GENDO project.

Inclusion Criteria	Exclusion criteria
Legal age (≥18years). Obesity class I or II with subjectively experienced weight-related impairment and a current intention to lose weight.	Obesity class III (i.e., BMI>39.9kg/m^2^).
Overweight (i.e., BMI between 25 and 29.9kg/m^2^) with weight-related health problems and/or visceral adipose tissue and/or high psychosocial weight-related distress with a current intention to lose weight.	Current (or within the last 12months) involvement in a structured weight loss intervention.
	Insulin-dependent type 1 diabetes.
	Previous or intended bariatric surgery.
	Current psychotherapeutic treatment of weight-related health problems.
	Weight-enhancing drugs.
	Drugs which promote weight-loss (e.g., anti-obesity drugs).
	Weight-enhancing health problems which are not yet treated.
	Cancerous disease within the last 5years.
	Current substance-use disorders, major depression, psychosis, suicidal tendency or pregnancy.
	Severe cognitive impairments.
	Insufficient knowledge of the German language.
	Binge-Eating-Disorder or Bulimia nervosa.

The final sample consisted of *N*=213 (67.1% female) individuals with obesity or overweight (M_BMI_=33.35kg/m^2^, SD_BMI_=3.79kg/m^2^). Age ranged between 19 and 71years (M_age_=46.45years, SD_age_=12.13years).

### Measures

#### Food Addiction (YFAS 2.0)

Addiction-like eating (during the past 12months) was measured using the YFAS Scale 2.0 (Gearhardt et al., 2016). The YFAS 2.0 consists of 35 items, which are scored on an 8-point scale ranging from *never* to *every day*. Based on the 11 diagnostic criteria for SUDs in the DSM-5 (e.g., craving, tolerance or withdrawal) a symptom score ranging between 0 and 11 is calculated, reflecting the FA symptom severity. Additionally, clinically significant impairments or distress due to the eating behaviour are assessed. Food addiction (FA) is diagnosed when two or more symptoms are met (=symptom score≥2) together with clinically significant impairments or distress due to the eating behaviour. The German version of the YFAS 2.0 was used (Meule et al., 2017) and showed excellent internal consistency, α=0.91, in the present sample.

#### Eating Disorders (Munich ED-Quest)

The Munich Eating and Feeding Disorder Questionnaire (Munich ED-Quest, Fichter et al., 2015) was used to screen for BN and BED. The whole questionnaire consists of 65 items assessing core symptoms of BN, BED, Anorexia Nervosa, Purging Disorder and Night Eating Syndrome within the last 3months. In our study, only 32 items were assessed screening for core symptoms of BED and BN based on the DSM-5 criterions (e.g., binge eating episodes, inappropriate compensatory behaviour, significant distress caused by binge eating episodes). Participants were instructed to indicate the severity of their symptomatology by either rating the corresponding items on a 5-Point Likert scale ranging from 0 to 4, or by estimating average frequencies (e.g., of binge eating episodes). Reliability of Munich ED-Quest (internal consistency of α=0.94) and validity are determined in a clinical sample of eating disorder patients in Germany (Fichter et al., 2015).

#### Depression (PHQ-9)

Depressive symptoms in the last 2weeks were measured using the Patients-Health-Questionnaire-9 (PHQ-9, Löwe et al., 2002). The screening instrument consists of nine items, corresponding to the DSM-IV major depression criteria, which are scored on a 4-point scale ranging from *not at all* (0) to *nearly every day* (3). The symptom score ranges between 0 and 27, with scores above 20 indicating major depression (Kroenke et al., 2001).

#### Alcohol Use Disorder (AUDIT)

The Alcohol Use Disorders Identification Test (AUDIT) was developed by the World Health Organization (WHO) to identify risky or harmful alcohol consumption, and alcohol use disorder (AUD; Saunders et al., 1993). The self-report questionnaire consists of 10 items. Each item is scored between 0 and 4. The sum score ranges between 0 and 40 points, with scores above the cut-off of 15 reflecting hazardous alcohol consumption (Conigrave et al., 1995). We used the German version of the AUDIT showing good psychometric properties in a German general practice population sample (Dybek et al., 2006). The internal consistency was adequate, α=0.68, in the present sample.

#### Internet Use Disorder (AICA-S)

The Assessment of Internet and Computer Game Addiction Scale (AICA-S, Wölfling et al., 2016) was used to measure problematic and pathological internet use. The AICA-S is a self-report questionnaire, consisting of 17 items, of which 14 are relevant for the clinical classification of pathological internet use based on the DSM-5-criteria for SUD. Scores of 13 or greater indicate internet use disorder (IUD). Reliability (internal consistency of α=0.89) and validity of the AICA-S are determined in the general population (Müller et al., 2014).

#### Psychological Distress (BSI-18)

The German short version of the Brief Symptom Inventory (BSI-18, Derogatis and Fitzpatrick, 2004) was used to assess psychological distress. The self-report questionnaire consists of 18 items which are rated on a five-point Likert scale ranging from *not at all* (0) to *always* (4). The total score indicates general distress (global severity index, GSI) and ranges between 0 and 72 points. The German translation of the scale showed good psychometric qualities in a sample of undergraduate students, non-clinical subjects and psychosomatic outpatients (Spitzer et al., 2011). The BSI-18 showed an excellent internal consistency, α=0.85, in the present sample.

#### Impulsivity (BIS-15)

To assess impulsivity as a personality trait, the short version of the German Barratt Impulsiveness Scale (BIS-15, Meule et al., 2011) was used. The self-report questionnaire consists of 15 items, which are scored on a four-point scale from *seldom/never* (1) to *almost always/always* (4). The overall sum score of impulsivity ranges between 15 and 60, with higher scores indicating higher degrees of impulsivity. In our study, the overall impulsivity trait was analysed. The internal consistency was good, α=0.80, in the present sample.

#### Impulsive Eating Behaviour (FEV)

The subscale *interference* of the Eating Behaviour Questionnaire (FEV, Pudel and Westenhöfer, 1989) was used to assess impulsive eating behaviour. The FEV_In consists of 16 items, each answered with either *true* (1) or *not true* (2). Three items are inverted (8, 10, and 12). The internal consistency was adequate, α=0.71, in the present sample.

#### Food Related Inhibitory Control (FRIS)

The Food Related Inhibitory Control Scale (FRIS, C. Seiferth and colleagues, not yet published) is a newly developed questionnaire, currently revised and validated, which consists of 40 items, each of it answered on a scale from *strongly disagree* (0) to *strongly agree* (5). The total score ranges between 0 and 200, with higher scores indicating increased food related inhibitory control. Preliminary validation proposes a four-factor solution with the subscales Action Withdrawal (AW, e.g., item: “I eat unconsciously between the meals,” Cronbach’s α=0.82), Action Cancellation (AC, e.g., item: “Once I started eating, I cannot stop anymore.,” Cronbach’s α=0.86), Reward Sensitivity (RS, e.g., item: “I reward myself with eating,” Cronbach’s α=0.69) and Delay Discounting (e.g., item: “If I get hungry, I chose rapidly available food,” Cronbach’s α=0.72).

#### Emotional Eating Behaviour (DEBQ)

The subscale *emotional eating* of the German translation of the Dutch Eating Behaviour Questionnaire (DEBQ, Van Strien et al., 1986) was used to measure emotional eating behaviour. The original self-report questionnaire consists of 30 items. The emotional eating subscale (DEBQ_EE) consists of 10 items, all answered on a five-point Likert scale ranging from *never* (1) to *very often* (5). The subscale score ranges between 10 and 50 points. Good psychometric properties of the German version of the scale have been demonstrated by Nagl and colleagues (Nagl et al., 2016). The internal consistency was excellent, α=0.92, in the present sample.

#### Weight Bias Internalization (WBI)

The German version of the Weight Bias Internalization Scale (WBIS, Hilbert et al., 2014) was used to measure internalized negative stereotypes and prejudice regarding overweight. The WBIS consists of 11 items which are rated on a seven-point scale ranging from *strongly disagree* (1) to *strongly agree* (7). The total score ranges between 11 and 77. The German version showed good psychometric properties (Hilbert et al., 2014). The internal consistency was good, α=0.86, in the present sample.

#### Self-Efficacy (SWE)

To measure self-efficacy, the General Self-Efficacy Scale (SWE, Jerusalem and Schwarzer, 2003) was used. The self-report questionnaire consists of 10 items, each of it answered on a four-point scale ranging from *not at all true* (1) to *exactly true* (4), yielding a total score between 10 and 40 points. The SWE showed good psychometric properties in a representative German sample (Hinz et al., 2006). The internal consistency was good, α=0.88, in the present sample.

### Statistical Analysis

All analyses were conducted with *IBM SPSS statistics for windows* (Version 26.0, Armonk, NY: IBM Corp.) and *Microsoft Excel* (Version 16.0, Microsoft Corporation). Descriptive analyses were conducted using percentages and frequencies for categorical variables, as well as means and standard deviations for continuous variables. *Chi-square* distributions that compared categorical variables between groups (FA vs. NFA) were implemented as well as Bonferroni-adjusted independent *t*-tests to compare metrically scaled variables. Associations between metrically scaled variables were analysed using *Pearson correlations*.

### Ethics

The study was carried out in accordance with the Declaration of Helsinki. The Institutional Review Board of the Ruhr-University Bochum approved the study (Nr. 18-6415). All subjects were informed about the study and all provided written informed consent.

## Results

### Prevalence of Food Addiction

The prevalence of *Food Addiction* was 15% (*N*=32), with higher, although not statistically significant, prevalence rates in female (18.2%, *N*=26) compared to male (8.6%, *N*=6) participants.

Table 2 gives an overview about sociodemographic factors both of the total sample and the subsamples of individuals experiencing food addiction (FA) or not experiencing FA (NFA). No differences between the subsamples (FA vs. NFA) regarding gender, age, BMI, marital status and education could be found.

**Table 2:** Sociodemographic factors (FA vs. NFA).

	Overall (*N*=213)	FA (*N*=32)	NFA (*N*=181)	FA vs. NFA
Sex	n_f_ =143, 67.1% n_m_ =70, 32.9%	n_f_ =26, 81.3% n_m_ =6, 18.8%	n_f_ =117, 64.6% n_m_ =64, 35.4%	χ^2^(1)=3.40, *p* =ns
Age (years)	M=46.45, SD=12.13	M=48.28, SD=12.14	M=46.12, SD=12.13	*t*(211)=0.93, *p* =ns
BMI (kg/m^2^)	M=33.35, SD=3.79	M=34.48, SD=3.27	M=33.15, SD=3.85	*t*(211)=1.84, *p* =ns
Marital Status^*^	n_yes_ =170, 79.8%	n_yes_ =26, 81.3%	n_yes_ =144, 79.6%	χ^2^(1)=0.05, *p* =ns
Education^**^	n_uni_ =61, 27.9%	n_uni_ =8, 25.0%	n_uni_ =53, 29.3%	χ^2^(1)=0.24 *p* =ns

FA, participants experiencing Food Addiction; NFA, participants not experiencing Food Addiction; n_f/m_, number of female/male participants; n_yes_, number of participants living in a relationship; n_uni_, number of participants who graduated university.

* Marital status: currently living in a relationship yes/no.

** Education: highest educational degree (in this case: university). n.s. = not significant

### Food Addiction, Psychopathology and Weight- and Addiction-Related Constructs

Correlation analyses revealed a significant positive association (*r*=0.17, *p*=0.014) between symptom severity of FA and BMI at baseline assessment (Table 3). With regard to psychopathology, FA symptom severity was positively correlated to severity of depressive symptoms (*r*=0.33, *p*<0.001), severity of IUD (*r*=0.18, *p*=0.011) and psychological distress (*r*=0.35, *p*<0.001). An impulsive (*r*=0.48, *p*<0.001) and emotional eating behaviour (*r*=0.36, *p*<0.001) as well as WBI (*r*=0.44, *p*<0.001) was positively associated with FA. There were negative correlations between FA symptom severity and the subscales action withdrawal (*r*=−49, *p*<0.001), action cancellation (*r*=−0.41, *p*<0.001), reward sensitivity (*r*=−0.46, *p*<0.001) and delay discounting (*r*=−0.27, *p*<0.001) of the FRIS. A decrease in self-efficacy was also associated with FA symptom severity (*r*=−0.21, *p*=0.002).

**Table 3:** Pearson correlations among psychological test scores (*N*=213).

S. No.		1.	2.	3.	4.	5.	6.	7.	8.	9.	10.	11.	12.	13.	14.	15.
1.	YFAS 2.0															
2.	Age (years)	0.036														
3.	BMI (kg/m^2^)	0.168^*^	−0.104													
4.	PHQ-9	0.334^**^	−0.225^**^	−0.046												
5.	AUDIT	−0.086	−0.030	−0.206^**^	0.037											
6.	AICA-S	0.175^*^	−0.237^**^	0.027	0.267^**^	−0.077										
7.	BSI-15	0.350^**^	−0.078	−0.049	0.563^**^	0.029	0.247^**^									
8.	BIS-18	0.056	0.035	0.093	0.128	0.005	0.168^*^	0.172^*^								
9.	FEV_In	0.478^**^	−0.158^*^	0.080	0.296^**^	−0.027	0.179^**^	0.352^**^	0.054							
10.	DEBQ_EE	0.356^**^	−0.135^*^	0.113	0.297^**^	−0.082	0.127	0.377^**^	−0.006	0.682^**^						
11.	WBIS	0.444^**^	−0.275^**^	0.268^**^	0.411^**^	−0.153^*^	0.236^**^	0.423^**^	0.121	0.460^**^	0.496^**^					
12.	SWE	−0.212^**^	−0.023	0.047	−0.323^**^	0.044	−0.089	−0.441^**^	−0.104	−0.273^**^	−0.265^**^	−0.311^**^				
13.	FRIS_AW	−0.487^**^	0.218^**^	−0.126	−0.212^**^	0.027	−0.282^**^	−0.201^**^	−0.037	−0.612^**^	−0.475^**^	−0.360^**^	0.137^*^			
14.	FRIS_AC	−0.407^**^	0.222^**^	−0.067	−0.211^**^	−0.108	−0.230^**^	−0.205^**^	−0.035	−0.620^**^	−0.404^**^	−0.338^**^	0.179^**^	0.708^**^		
15.	FRIS_RS	−0.461^**^	0.178^**^	−0.102	−0.205^**^	0.076	−0.195^**^	−0.213^**^	0.075	−0.636^**^	−0.620^**^	−0.416^**^	0.155^*^	0.626^**^	0.592^**^	
16.	FRIS_DD	−0.274^**^	0.110	−0.077	−0.237^**^	0.168^*^	−0.191^**^	−0.161^*^	−0.005	−0.341^**^	−0.341^**^	−0.282^**^	0.150^*^	0.389^**^	0.338^**^	0.447^**^

YFAS, Yale Food Addiction Scale; PHQ-9, Patients-Health-Questionnaire-9 (depression); AUDIT, Alcohol Use Disorders Identification Test; AICA-S, Assessment of Internet and Computer Game Addiction Scale; BSI-15, Brief Symptom Inventory (Psychological Distress); BIS-18, Barratt Impulsiveness Scale (Impulsivity Personality Trait); FEV_In, Eating Behaviour Questionnaire (Subscale: Interference); DEBQ_EE, Dutch Eating Behaviour Questionnaire (subscale: Emotional Eating); WBIS, Weight Bias Internalization Scale; SWE, General Self-Efficacy Scale; FRIS, Food Related Inhibitory Control Scale (Subscales: AW, Action Withdrawal; AC, Action Cancellation; RS, Reward Sensitivity; DD, Delay Discounting).

* Correlation is significant at the 0.05 level (2-tailed);

** Correlation is significant at the 0.01 level (2-tailed).

Table 4 illustrates results from independent t-Tests between the subsamples (FA vs. NFA) on psychosocial measurements and psychopathology. FA participants showed significantly higher FA symptom severity (YFAS 2.0) than NFA participants did. Additionally, the GSI (BSI-15) was higher for FA compared to NFA participants, indicating higher psychological distress. Comparisons of psychosocial measurements associated with eating behaviour indicated that FA participants suffer from a more impulsive (FEV_In) and emotional eating behaviour (DEBQ_EE), as well as weight bias internalization (WBIS). Moreover, FA participants scored less on three of four FRIS subscales (AW, AC and RS), indicating impairments in behavioural inhibitory control and increased reward sensitivity. The groups did not differ on the subscale DD, reflecting impulsive choices.

**Table 4:** Psychopathology and weight- and addiction-related constructs (FA vs. NFA).

	Total (*N*=213)		FA (*N*=32)		NFA (*N*=181)					FA vs. NFA
	M	SD	M	SD	M	SD				
YFAS 2.0	2.46	2.57	5.81	2.69	1.86	2.04	t(211)=7.91	*p* <0.001	d=1.84	FA>NFA
PHQ-9	6.17	3.96	7.97	4.20	5.86	3.84	t(211)=2.83	*p* =n.s.		
AUDIT	4.23	3.20	3.59	3.43	4.34	3.16	t(211)=1.21	*p* =n.s.		
AICA-S	4.46	2.96	5.00	4.14	4.37	2.70	t(211)=0.83	*p* =n.s.		
BSI-18	8.44	7.01	12.81	8.92	7.66	6.34	t(211)=3.13	*p* =0.042	d=0.76	FA>NFA
BIS-15	30.85	5.96	30.50	6.38	30.91	5.89	t(211)=0.36	*p* =n.s.		
FEV_In	9.69	3.24	11.31	2.95	9.41	3.22	t(211)=3.12	*p* =0.028	d=0.60	FA>NFA
DEBQ_EE	3.06	1.02	3.70	0.85	2.94	1.01	t(211)=4.01	*p* <0.001	d=0.71	FA>NFA
WBIS	44.08	13.31	54.84	11.41	42.17	12.74	t(211)=5.27	*p* <0.001	d=1.01	FA>NFA
SWE	28.71	4.30	28.72	4.93	28.71	4.19	t(211)=0.007	*p* =n.s.		
FRIS_AW	30.28	10.58	24.34	9.11	31.33	10.49	t(211)=3.54	*p* <0.001	d=0.68	FA<NFA
FRIS_AC	29.09	9.97	24.22	11.29	29.96	9.49	t(211)=3.06	*p* =0.042	d=0.59	FA<NFA
FRIS_RS	11.72	4.98	8.81	5.36	12.24	4.74	t(211)=3.69	*p* <0.001	d=0.71	FA<NFA
FRIS_DD	12.38	3.90	11.03	3.88	12.62	3.87	t(211)=2.14	*p* =n.s.		

Bonferroni-adjusted *p* values, effect size Cohen’s d. n.s = not significant

## Discussion

The main aim of the present study was to assess the prevalence of FA in a treatment-seeking sample of individuals with overweight or obesity and without comorbid BN or BED as previous studies did not control for these comorbid disorders. The prevalence of FA in our sample was 15%, with higher, although not statistically significant, rates in female (18.2%) compared to male participants (8.6%) as expected (Pursey et al., 2014). Notably, the higher prevalence rates in female participants might also be explained by a general tendency of women to over-report health problems compared to men (Boerma et al., 2016). Our findings fit the assumption that the prevalence of FA is higher in individuals with overweight and obesity (17.2%) compared to the general population (7.9%; Pursey et al., 2014; Hauck et al., 2017). Previous studies analysing FA in WLP samples, in which participants suffering from BED and BN were not excluded from participation, reported prevalence rates ranging between 15 and 38% (Eichen et al., 2013; Meule et al., 2015). The lower prevalence rate in our study might therefore be due to the fact, that individuals suffering from BN or BED were excluded from participation. Still, since 15% of our sample experienced FA, we thus conclude that, even when adjusting for comorbid eating disorders, a relevant subgroup of WLP participants experiences an addiction-like eating behaviour.

FA severity was associated with increased BMI, which is in line with findings of previous studies (Pedram et al., 2013; Gearhardt et al., 2014). A variety of studies reports associations between FA and SUDs (Davis and Carter, 2009; García-García et al., 2014; Tinghino et al., 2020). In our study, AUD severity was not associated with FA. This might be because hazardous alcohol consumption was an exclusion criterion for participation, but the missing correlation could also be masked by the inverse relationship between AUD and BMI. However, in line with previous studies, FA was associated with severity of depressive symptoms and psychological distress (Gearhardt et al., 2012; Burmeister et al., 2013). Although participants meeting the diagnostic criteria for major depression and AUD were excluded from participation, our results underline the strong relationship between FA symptomatology and mood disorders, and enlighten the high level of distress in individuals experiencing FA, which together might contribute to weight gain (Bourdier et al., 2018). FA was linked to IUD severity. IUD is a behavioural addiction characterized by excessive internet usage and an impaired control over usage behaviour (Young, 1996). Excessive internet usage is associated with unhealthy eating and sedentary behaviour and might subsequently aggravate FA symptomatology and contribute to weight gain (Vandelanotte et al., 2009; Yildirim et al., 2018).

Moreover, FA was linked to emotional eating and WBI and both were significantly higher in FA compared to NFA participants. There seems to be a shift from positive reinforcement to negative reinforcement in individuals experiencing FA regarding the consumption of palatable food (Parylak et al., 2011). Given that palatable food can be both rewarding and stress reducing, food deprivation may lead to withdrawal (i.e., stress enhancement) and a subsequent shift from gratification to compulsive eating. In line with previous studies, self-efficacy was negatively correlated to FA symptom severity in our sample (Burmeister et al., 2013; Cassin et al., 2019). Low self-efficacy and high WBI are linked to lower physical activity and WBI predicts reduced odds of achieving weight-loss in individuals with overweight and obesity (Hübner et al., 2015; Pearl et al., 2019). Therefore, the reported associations between FA, WBI and self-efficacy fits prior observations that individuals experiencing FA suffer from weight cycling (Gearhardt et al., 2014). It is assumed that the relationship between WBI and FA is moderated by emotional dysregulation, which should be further investigated (Baldofski et al., 2016). Future studies might also consider assessing weight control or eating self-efficacy instead of global self-efficacy (Linde et al., 2004; Cassin et al., 2019).

FA was associated with an impulsive eating behaviour and deficits in food-related inhibitory control. FA participants significantly differed from NFA participants in food-related impulsive actions (action withdrawal and cancelation), as well as reward sensitivity. This indicates that individuals experiencing FA have more problems in resisting impulses towards disposable and rewarding food and might therefore need specific training elements to enhance weight loss (Meule, 2019a). Delay discounting was associated with FA symptom severity, but the subgroup of FA participants did not significantly differ from NFA participants on this subscale. This might be due to the fact that delay discounting is generally increased in individuals with obesity (Mole et al., 2015). Interestingly, FA was not associated with the impulsivity personality trait. This is in contrast to prior studies indicating that FA, as well as other SUDs and EDs are associated with higher impulsivity traits (Mobbs et al., 2010; Churchill and Jessop, 2011; Murphy et al., 2014; VanderBroek-Stice et al., 2017). Since BN, BES and AUD were exclusion criteria in our study, it might be possible, that overlapping symptoms with this disorders confounded prior results. Based on our results, we hypothesize that not a global disposition towards impulsive behaviour, but rather a learning process that food is rewarding and disposable, may contribute to the impulsive eating behaviour in FA participants. In our study, impulsivity personality trait, impulsive eating behaviour and food-related inhibitory control were measured using self-reporting questionnaires. Since self-reported and behavioural results regarding disinhibition of eating behaviour in individuals with obesity can differ, the results of our study should be further analysed using behavioural tasks (Loeber et al., 2012). Furthermore, potential moderating factors like restrained eating or current mood should be considered (Loeber et al., 2018).

A particular strength of our study is that we analysed FA in a sample of individuals with overweight and obesity who did not suffer from eating disorders, which was verified by questionnaire and structured interview (*two stage design*). However, when interpreting the findings of the present study a few limitations should be acknowledged. Individuals with obesity class III (BMI>39.9kg/m^2^) were excluded from participation due to the study design of the WLP. It can be assumed that the prevalence of FA would have been higher when including these participants, since FA is reported to be increased in individuals experiencing extreme obesity (ca. 30%; Hauck et al., 2017). In addition, since AUD and major depression were exclusion criteria, the reported associations between FA, AUD severity and severity of depressive symptoms might be confounded. Since the risk for FA increases in polyabusers, the exclusion of individuals suffering from hazardous alcohol consumption might subsequently cause a lower prevalence of FA in our study (Tinghino et al., 2020). Finally, a broad test battery, i.e., with regard to different components of impulsivity and impulsive eating behaviour, was used. Still, our conclusions are based on results from self-reporting questionnaires and different results may be observed when using behavioural measurements as for example reported by Loeber et al. (2018). In addition, typical pre-test self-report biases are known, which might have further influenced our results (Aiken and West, 1990). Yet, despite its limitations, the YFAS is currently the “state-of-the-art” assessment of FA (Schulte et al., 2020). With a view to a potential inclusion of FA in the diagnostic catalogues, it would make sense to develop and use objective diagnostic assessment tools, like structured interviews (Gearhardt and Hebebrand, 2021; Hebebrand and Gearhardt, 2021). The interpretation of our results is aggravated due to our cross-sectional study design, i.e., with regard to causal analyses. It would subsequently be meaningful to verify the results in longitudinal studies, i.e., with regard to long-term weight management in individuals experiencing FA.

Summing up, our results support the view that, even when adjusting for BN and BED, a relevant subgroup of individuals with overweight and obesity experiences an addiction-like eating behaviour. This subgroup differs from non-addictive eaters on several weight- and addiction-related factors, like emotional eating, WBI, and impulsivity. Moreover, individuals experiencing FA suffer from depressive symptoms, addictive disorders and psychological distress. In sum, these impairments may contribute to weight gain. If so, our results underline that the lack of an officially approved FA diagnosis might currently cause an insufficient clinical care for individuals experiencing an addiction-like eating behaviour. It might therefore be reasonable to investigate the effect of FA on weight loss when adjusting for eating disorders and to further implicate addiction-specific therapeutic elements in WLPs to enhance weight loss and prevent weight regain in this subgroup.

## Data Availability Statement

The datasets presented in this article are not readily available because data will be made available only on reasonable request. Requests to access the datasets should be directed to magdalena.pape@rub.de.

## Ethics Statement

The studies involving human participants were reviewed and approved by The Institutional Review Board of the Ruhr-University Bochum (Nr. 18-6415). The patients/participants provided their written informed consent to participate in this study.

## Author Contributions

MP: conceptualisation, acquisition of data, formal analysis, interpretation of data, and writing – original draft. SH: conceptualisation, writing- original draft, and study supervision. SS and CS: study design, acquisition of data, and review and editing. TF: acquisition of data and review and editing. JW: study design, study supervision, and review and editing. SS-L: study design and conceptualisation, study supervision, and review and editing. All authors contributed to the article and approved the submitted version.

## Funding

The Federal Ministry of Education and Research, Germany (BMBF) funded the study.

## Conflict of Interest

The authors declare that the research was conducted in the absence of any commercial or financial relationships that could be construed as a potential conflict of interest.

## Publisher’s Note

All claims expressed in this article are solely those of the authors and do not necessarily represent those of their affiliated organizations, or those of the publisher, the editors and the reviewers. Any product that may be evaluated in this article, or claim that may be made by its manufacturer, is not guaranteed or endorsed by the publisher.
